# Lithium, the gold standard drug for bipolar disorder: analysis of current clinical studies

**DOI:** 10.1007/s00210-024-03210-8

**Published:** 2024-06-25

**Authors:** Magdalena Airainer, Roland Seifert

**Affiliations:** Institute of Pharmacology, Hannover Medical School, Carl-Neuberg-Straße 1, D-30625 Hannover, Germany

**Keywords:** Lithium, Bipolar disease, Mania, Depression

## Abstract

Lithium is the gold standard drug in the treatment of bipolar disorder. Despite increasing scientific interest, relatively few patients with bipolar disorder receive lithium therapy. Lithium is the only drug that is effective in the prophylaxis of manic, depressive, and suicidal symptoms. Lithium therapy is also associated with a variety of adverse drug reactions and the need for therapeutic drug monitoring. Numerous studies have focussed on the efficacy and safety of both lithium-monotherapy and lithium-add-on therapy. The aim of this study is to provide a systematic overview of clinical studies on lithium therapy for bipolar disorder from the last 7 years and to present a critical analysis of these studies. The results provide an up-to-date overview of the efficacy, tolerability, and safety of lithium therapy for bipolar disorder and thus improve the pharmacotherapy of bipolar disorder. A total of 59 studies were analysed using various analysis parameters. The studies were also categorised into different subgroups. These are lithium-monotherapy, lithium vs. placebo/drug, and lithium + adjunctive therapy. The majority of the studies (*N* = 20) had a duration of only 3–8 weeks. Only 13 studies lasted for > 40 weeks. Lithium was superior to aripiprazole, valproic acid, and quetiapine in terms of improving manic symptoms. Lithium therapy resulted in a lower relapse rate compared to valproic acid therapy. Lithium was more neuroprotectively effective than quetiapine. Fourteen of the 22 add-on therapies to lithium showed a predominantly positive effect on the treatment outcome compared to lithium-monotherapy. Only the add-on therapy with sertraline led to a higher rate of study discontinuations than lithium-monotherapy. Lithium is a safe and effective treatment option for children. However, risperidone and quetiapine were superior to lithium in some aspects, which is why these drugs should be considered as an alternative treatment option for children. Collectively, current clinical studies highlight the relevance of lithium in the treatment of bipolar disorder.

## Introduction

Around 0.5% of the world’s population suffer from bipolar disorder. (https://de.statista.com/statistik/daten/studie/1078901/umfrage/anteil-der-weltbevoelkerung-mit-bipolarer-stoerung/#:~:text=In%20year%202019%20suffered%20worldwide,%20about%20200%2C5%20percent; last call: 10.12.2023). It is characterised by alternating manic and depressive episodes. Depending on the manifestation, a distinction is made between bipolar-1-disorder and bipolar-2-disorder (https://www.msdmanuals.com/de-de/heim/psychische-gesundheitsstörungen/affektive-störungen/bipolare-störung; last call: 10.12.2023).

Lithium is considered the gold standard drug for the treatment of bipolar disorder (Seifert, Roland: Basiswissen Pharmakologie, 2nd edition, Germany, Springer-Verlag GmbH, 2021, p. 397). Lithium therapy is associated with a variety of adverse drug reactions and a narrow therapeutic range, which requires therapeutic drug monitoring (Seifert, Roland: Basiswissen Pharmakologie, 2nd edition, Germany, Springer-Verlag GmbH, 2021, pp. 396–397). Subtherapeutic doses of lithium have positive effects on metabolic, cognitive, cardiovascular, and some other functions (Hamstra et al. 2023). This could be used as an approach for future studies investigating possible positive effects of low lithium doses on bipolar disorder.

An early start of therapy with lithium can have a positive influence on the course of the disease. In particular, lithium halted the reduction of white matter and improved neuronal connections between the striatum and the cerebellum compared to quetiapine (Rybakowski 2018). However, too few patients suffering from bipolar disorder are treated with lithium (Poranen et al. 2022). Patients who have taken lithium for almost 50 years have achieved very positive therapy results. The patients were all good lithium responders and the therapy with lithium was started in the early phase of the disease. These data epitomise the efficacy of lithium in bipolar disorder (Ferensztajn-Rochowiak et al. 2024).

Various mechanisms of action of lithium are being discussed. These include, in particular, GSK-3β. However, the exact mechanism of action of lithium is unknown (Mohamadian et al. 2023).

**Fig. 1:**
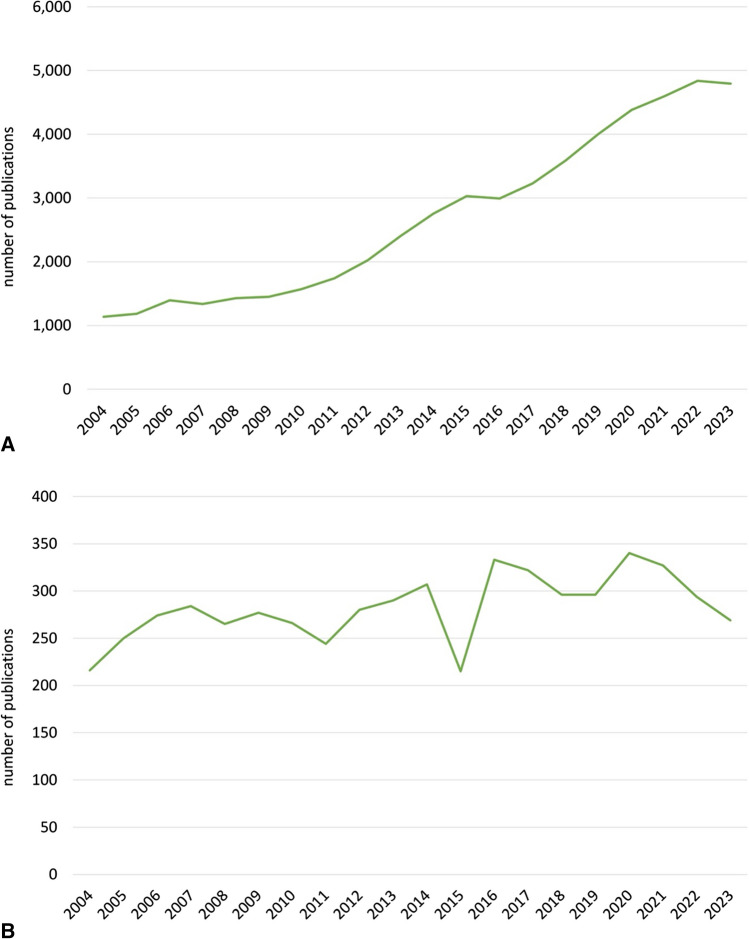

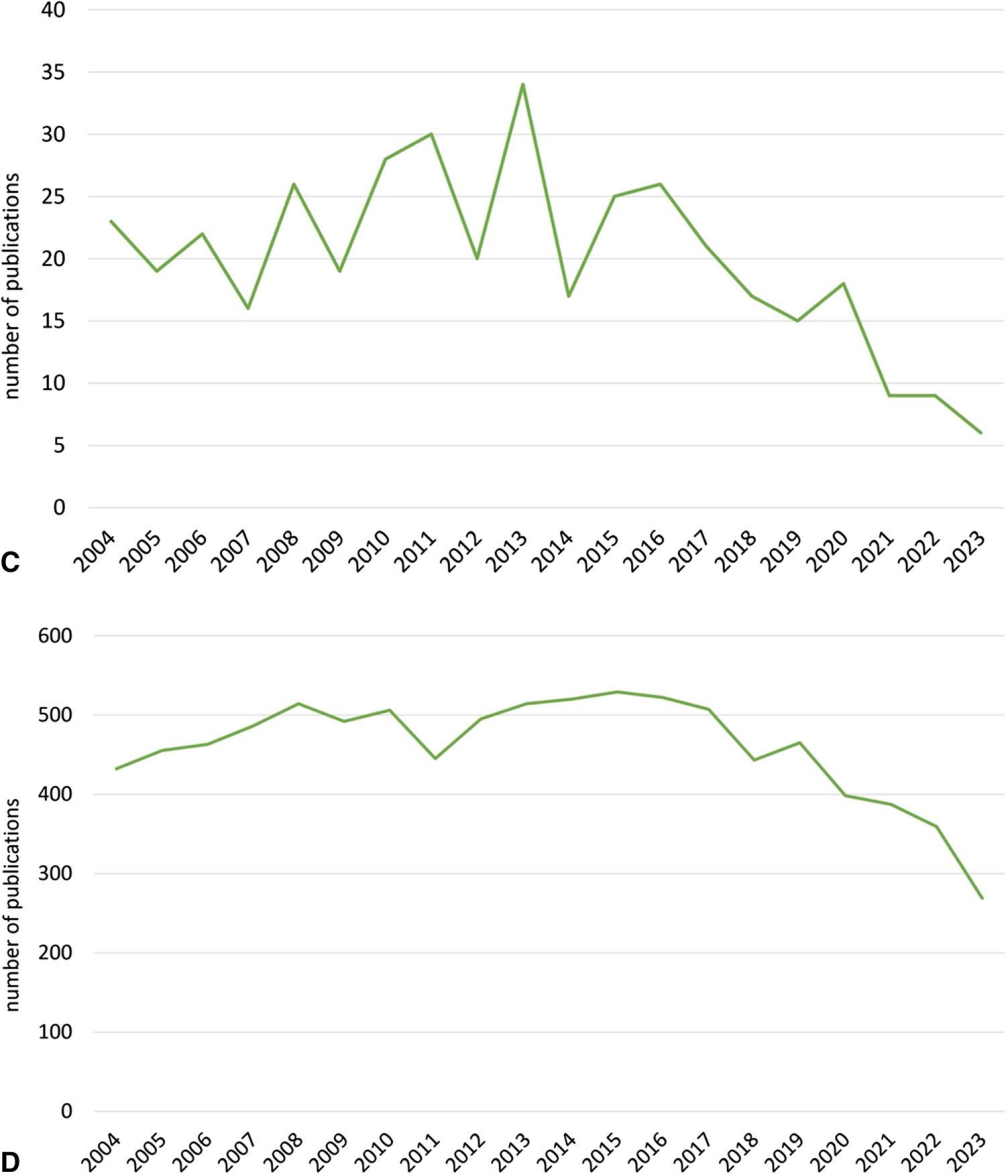

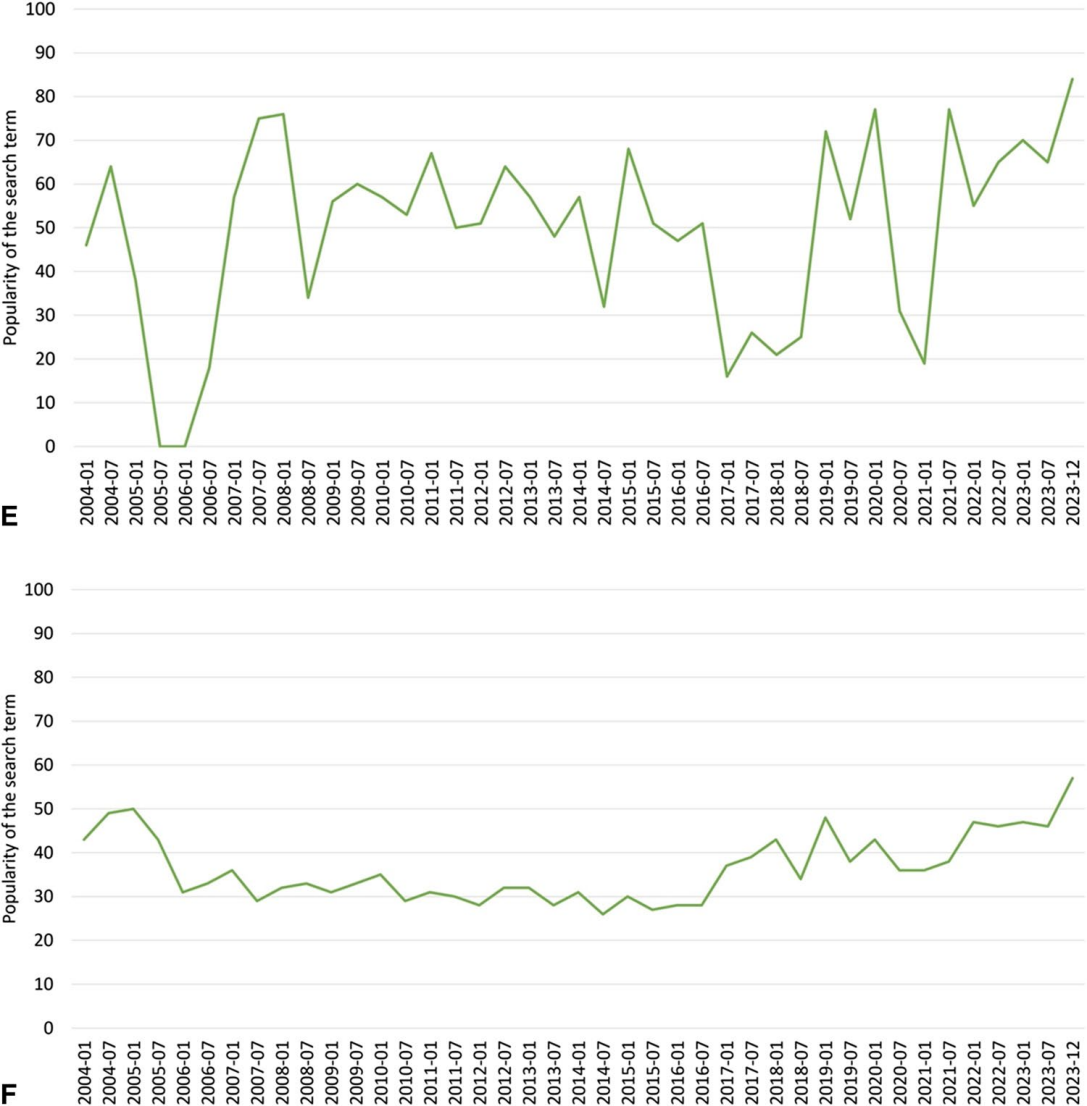
**A** Time trend on PubMed for “lithium”, 2004–2023 (https://pubmed.ncbi.nlm.nih.gov/?otool=idemhhlib, accessed: 13. December 2023). **B** Time trend on PubMed for “lithium AND bipolar disorder”, 2004–2023 (https://pubmed.ncbi.nlm.nih.gov/?otool=idemhhlib, accessed: 13. December 2023). **C** Time trend on PubMed for “lithium AND G-protein-coupled receptors”, 2004–2023 (https://pubmed.ncbi.nlm.nih.gov/?otool=idemhhlib, accessed: 13. December 2023). **D** Time trend on PubMed for “lithium AND mechanism of action”, 2004–2023 (https://pubmed.ncbi.nlm.nih.gov/?otool=idemhhlib, accessed: 13. December 2023). **E** Google search timeline (Germany), 2004–2023 half-yearly (https://trends.google.com/trends/?hl=de; accessed: 13. December 2023). **F** Google search timeline (worldwide), 2004–2023 half-yearly (https://trends.google.com/trends/?hl=de; accessed: 13. December 2023)

In recent years, scientific interest in “lithium” has increased (Fig. 1A). With regard to “Lithium and bipolar disorder”, “Lithium and G-protein-coupled receptors”, and “Lithium mechanism of action”, the number of PubMed publications has tended to fall (Fig. 1B–D). This could be due to the fact that, in the sense of “repurposing”, new possible indications for lithium therapy, such as Alzheimer’s dementia or brain tumours, are scientifically more interesting than the original indications, for which there is already proof of efficacy. The interest in society as a whole in lithium has remained relatively constant in recent years, both globally and specifically in Germany. This can be deviated from the interest in lithium on Google (Fig. 1E, F).

This paper is intended to provide a systematic overview of clinical studies on lithium therapy for bipolar disorder over the last 7 years and to critically analyse these studies. The main aim is to show which add-on therapies to lithium are useful and whether these are associated with better therapeutic outcomes compared to monotherapy. Another important issue will be the comparison of lithium with other drugs. The main aim here is to demonstrate for which symptoms lithium was more or less effective than other drugs. Furthermore, clinical predictors could make it possible to predict the success of treatment with lithium. Studies on paediatric patients will be presented separately in this analysis.

## Methods

### Data used

**Fig. 2:**
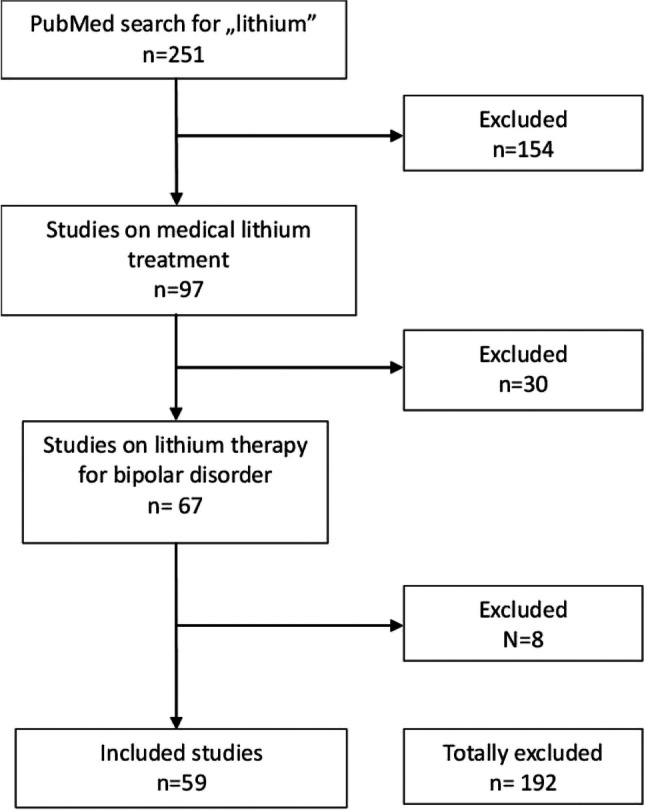
The filtering process of all analysed studies

**Fig. 3:**
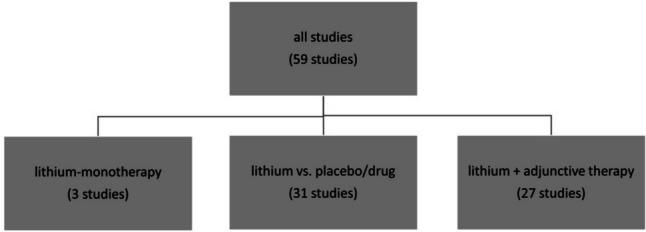
Depiction of the division of studies into subgroups

The included studies were listed in the PubMed database (https://pubmed.ncbi.nlm.nih.gov/?otool=idemhhlib) in the period 2015–2022. For the search term “lithium” with filters for randomised clinical trials and clinical trials, 251 results were found. The filtering process of the studies is shown in Fig. 2. As a first step, all studies that did not deal with lithium therapy in humans were excluded (*N* = 154). From the remaining *N* = 97 studies, all studies dealing with lithium therapy for bipolar disorder were filtered out. The prerequisite was that patients with bipolar-1-disorder or bipolar-2-disorder received lithium-monotherapy or an add-on therapy to lithium. In a final step, studies that had not yet been completed or in which lithium therapy only played a secondary role were excluded (*N* = 8). The 59 eligible studies were analyzed in Excel tables and categorised into different subgroups. These are lithium-monotherapy (*N* = 3), lithium vs. placebo/drug (*N* = 31), and lithium + adjunctive medication (*N* = 27) (Fig. 3). Some studies were assigned to more than one subgroup. Studies in paediatric patients were analysed separately. The studies (*N* = 10) that analysed patients aged < 18 years were used for this purpose. The 10 largest studies with participant numbers > 300 were also considered separately, as they can be assumed to have the greatest clinical relevance.

### Analyses carried out

The following parameters were analyzed: initial diagnosis of the study participants, disease episode of the study participants, type of randomisation, control group yes/no, number of participants, age range of the study participants, mean age of the study participants, duration of the study, conflict of interest yes/no, sponsor, country of study conduct, journal, and study type. For the initial diagnosis of the study participants, a distinction was made between bipolar-1-disorder, bipolar-2-disorder, and bipolar-1- or bipolar-2-disorder. For the episode of the disease, a distinction was made between “mania”, “depression”, and “every phase” (mania and depression). By type of randomisation, studies were categorised as “double-blind”, “single-blind”, “open-label”, or “no randomisation”. If no information on the type of randomisation was provided, the studies were assigned to the category “not specified”. Under the analysis item “control group yes/no”, studies were categorised as “yes, placebo” if there was a placebo control group, “yes, other drug” if the control group received a different medication, “yes, placebo and other drug” if there was both a placebo control group and a control group with a different medication, and “no” if there was no control group. The number of participants, duration of the study, mean age and age range were used as stated in the studies. Studies were counted as “conflict of interest yes” whenever one of the authors reported a conflict of interest. Studies in which none of the authors had a conflict of interest were counted as “conflict of interest no”. All sponsors of the studies listed in the study were considered under “sponsoring”. The country in which the study was conducted was determined on the basis of the locations specified in the study. For localisations in more than one country, the study was assigned to “international”. When analysing the study type, a distinction was made between “RCT” (randomised clinical trial) and “CT” (clinical trial).

The acquired data were presented graphically to identify trends and facilitate comparisons. All graphs were created using Excel. Further analyses were carried out. These included the determination of defined daily dose costs and defined daily dose over time. These data were obtained from the “Drug Prescription Reports” (Arzneiverordnungsreport, AVR) for the statutory health insurance in Germany for the years 1987–2022. The defined daily doses (DDD) for 2021 were used to calculate approximately how many patients with bipolar disorder received lithium therapy. This was based on a German population of 83.2 million, a 12-month prevalence of bipolar disorder of 0.29% and 90% of patients with statutory health insurance. In addition, the studies from the subgroup “lithium + add-on therapy” were checked for the methodology used with the aid of a scoring system.

## Results and discussion

### All studies

**Fig. 4:**
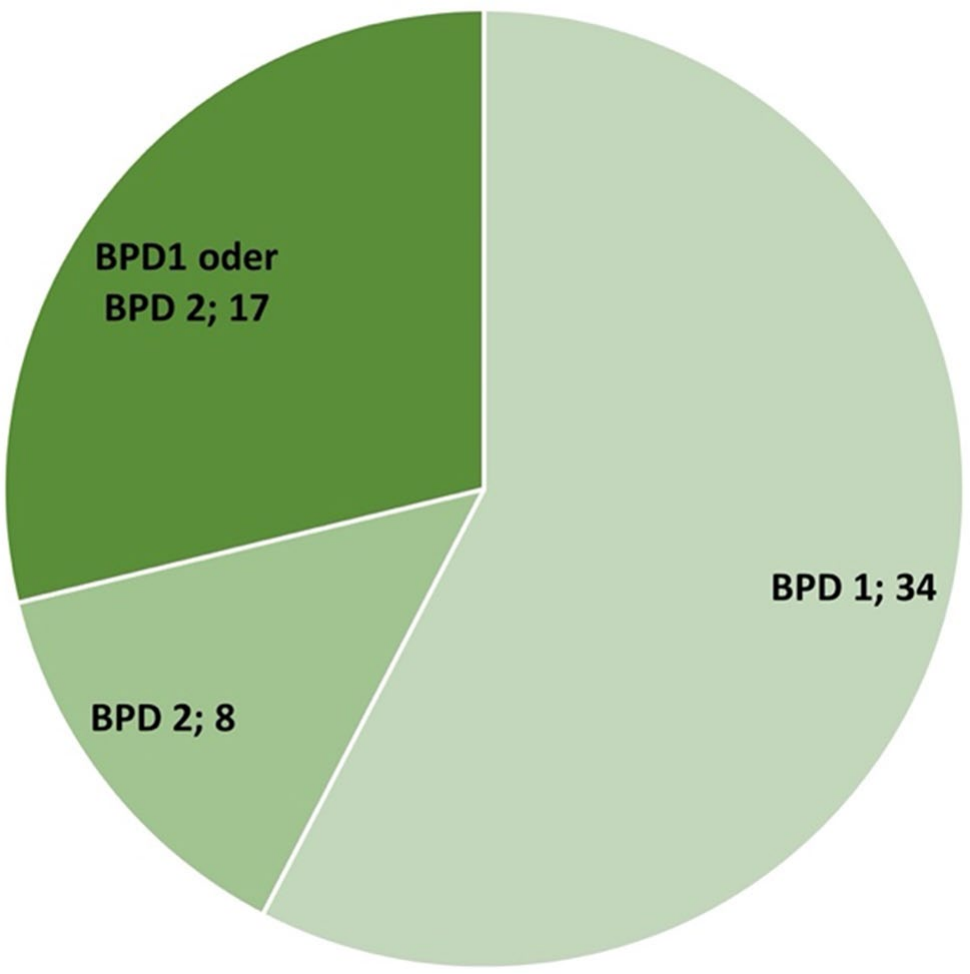
Baseline diagnosis of study participants of all studies

**Fig 5:**
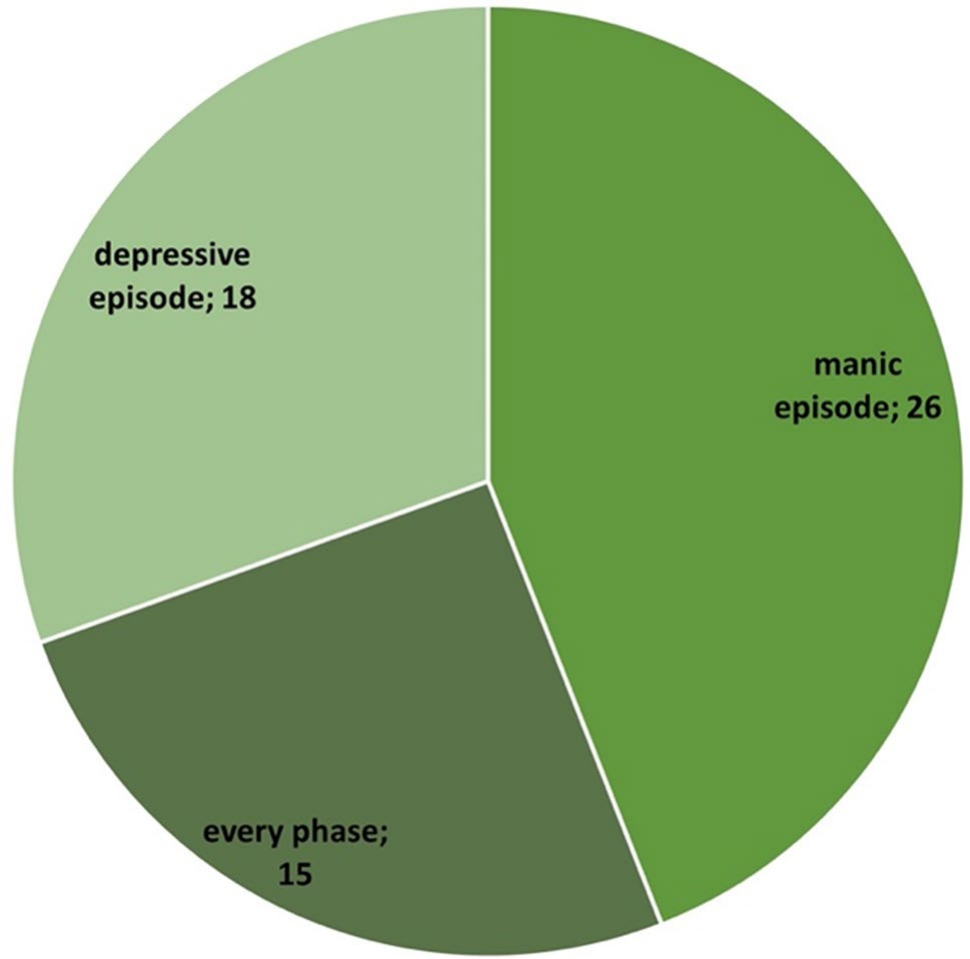
Disease episode of the study participants of all studies

**Fig. 6:**
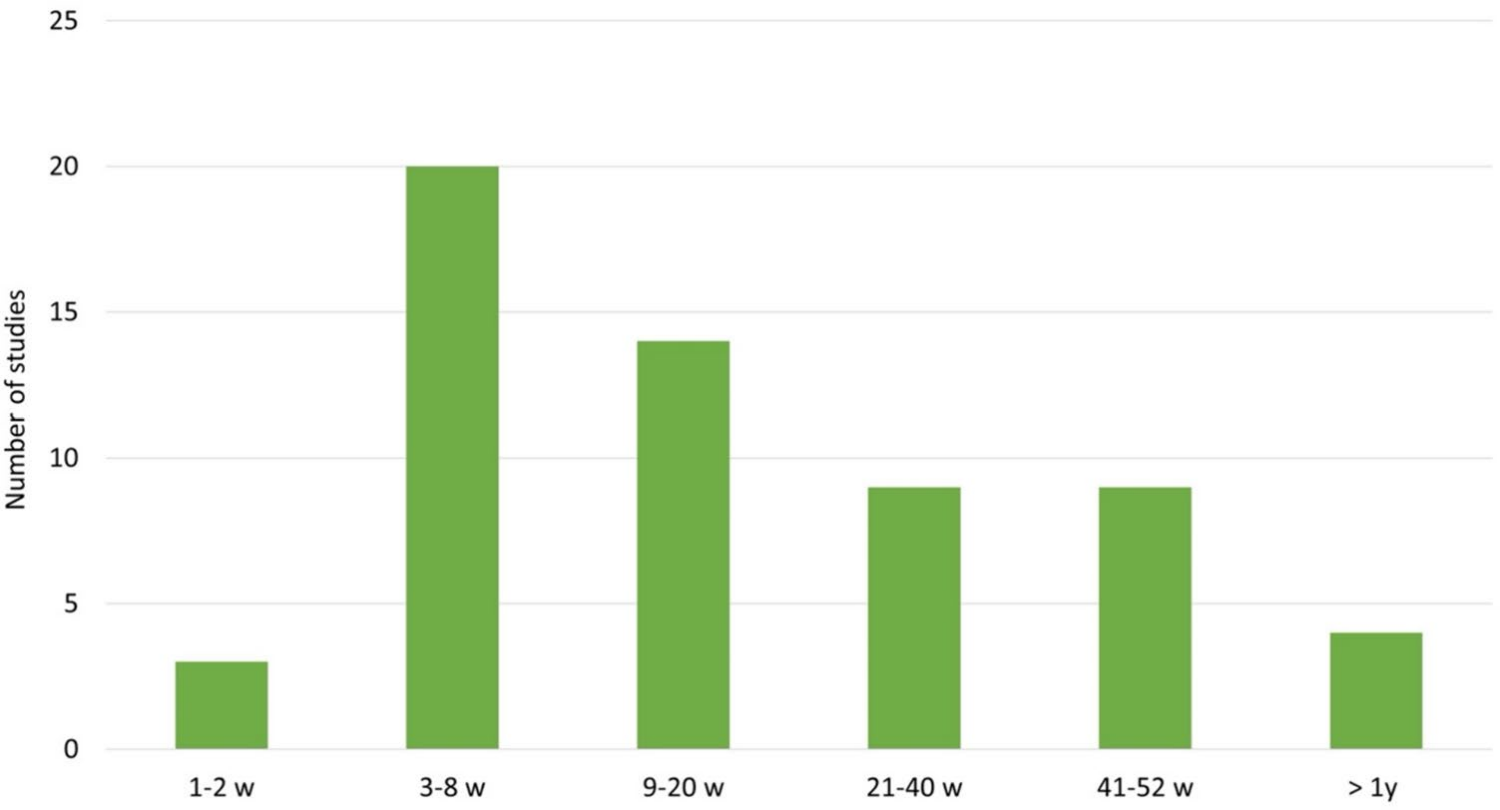
Duration of studies in weeks

**Fig. 7:**
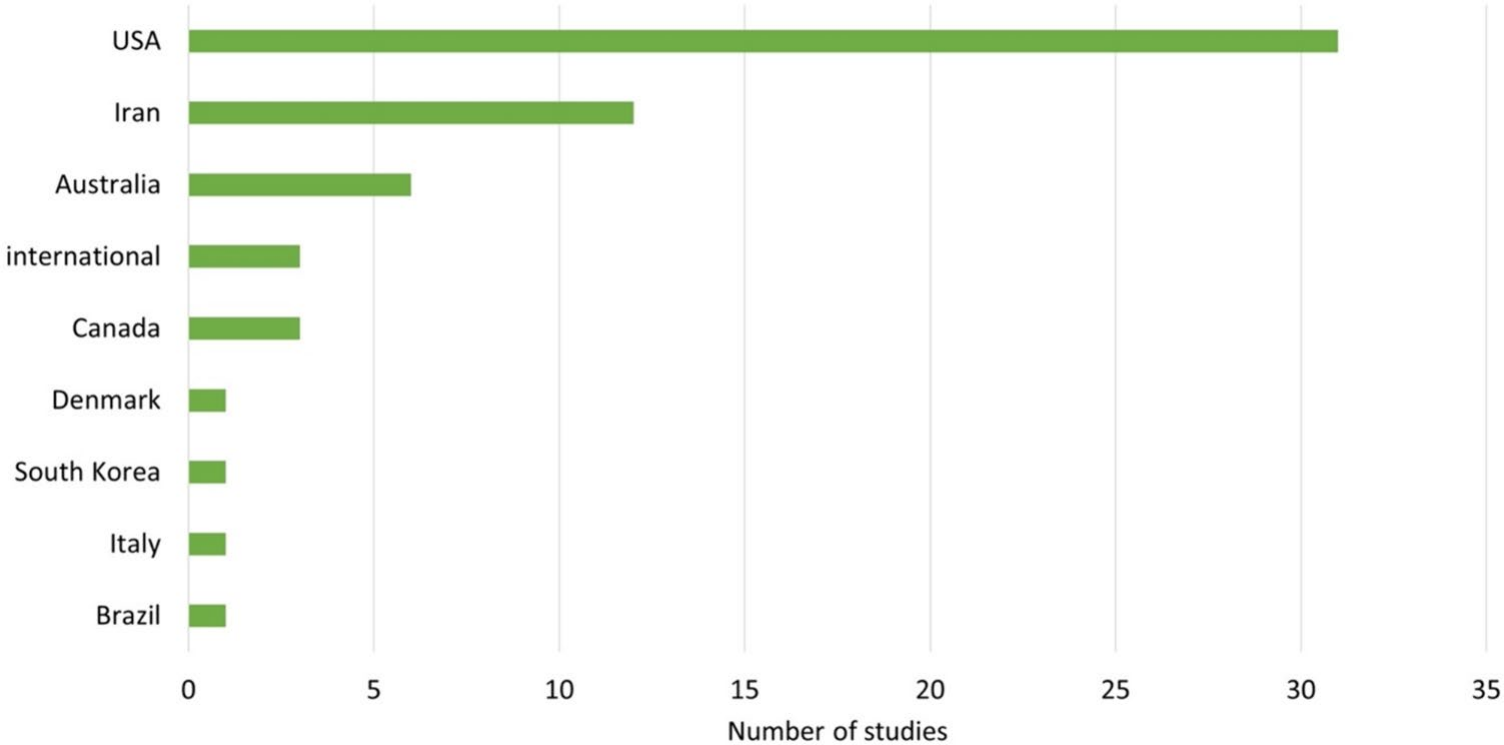
Country of study conduct

**Fig. 8:**
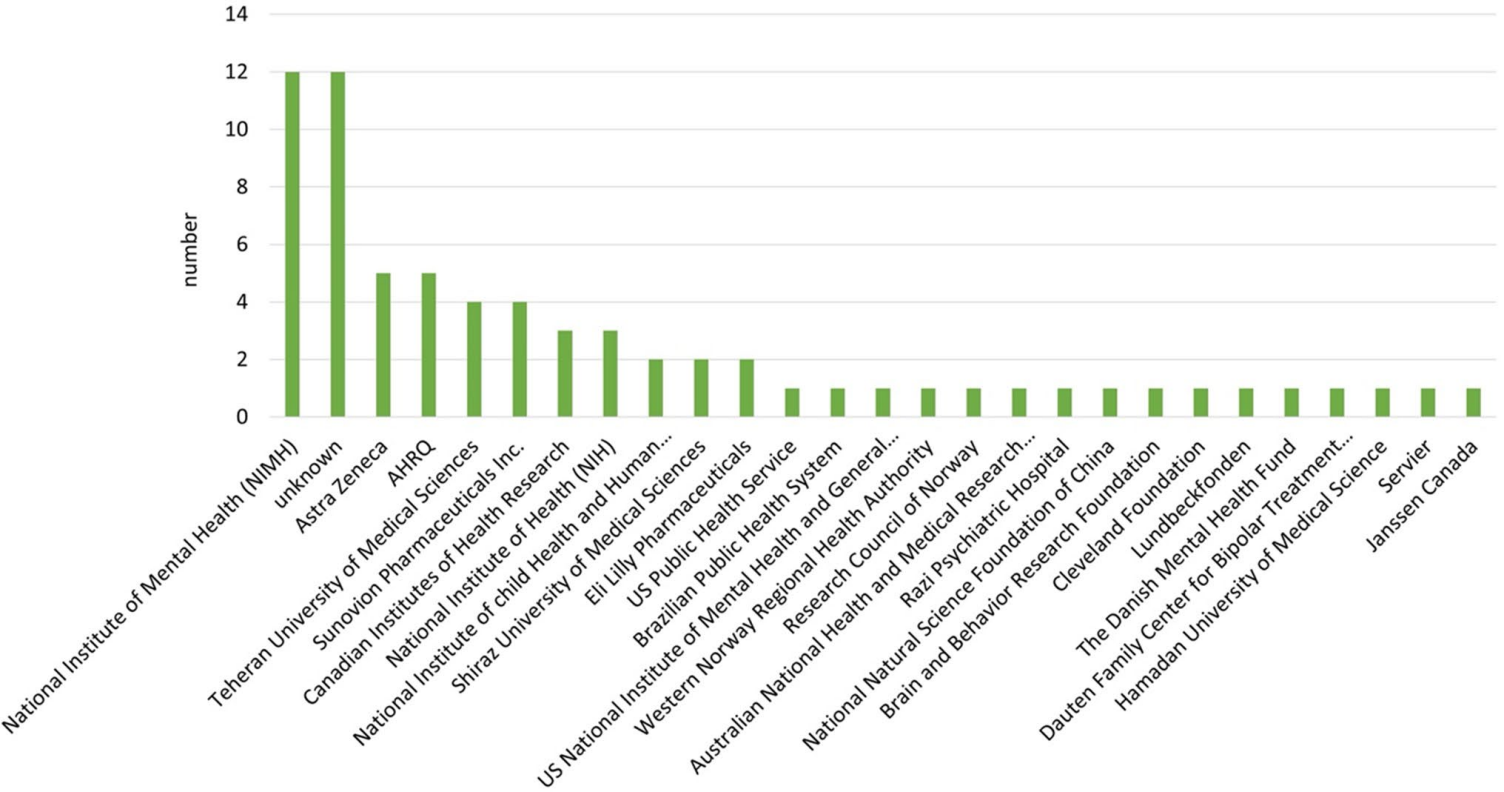
Sponsoring of the studies

Among the studies analysed, there were more studies on patients with bipolar-1-disorder (*N* = 34; 58%) than on patients with bipolar-2-disorder (*N* = 8; 13%). In some of the studies, it was not specified which form of bipolar disorder (bipolar-1- or bipolar-2-disorder) the patients were required to have in order to be included in the study (*N* = 17; 29%) (Fig. 4). When analysing the episode of illness of the study participants, there were *N* = 26 studies on patients in the mania episode and *N* = 18 studies on patients in a depressive episode (Fig. 5). The majority of the studies (*N* = 20) had a duration of only 3–8 weeks. Only *N* = 13 of *N* = 59 of the studies analysed involved monitoring patients over a period of > 40 weeks. Three studies ran for a period of only 1–2 weeks (Fig. 6). Most of the studies were conducted in the USA (*N* = 31), *N* = 12 studies in Iran. In addition, *N* = 2 studies were conducted in European countries (Denmark and Italy). In total, the studies are from eight different countries (Fig. 7). Almost all studies were funded by public institutions and foundations with no commercial interest (Fig. 8). Companies involved in sponsoring produced the additive or alternative drug in the studies concerned.

### Focus on bipolar-1-disorder

The fact that there are more studies on patients with bipolar-1-disorder among the analysed studies (Fig. 4) cannot be explained by the respective prevalence. Patients in a severe episode of mania experience serious social and financial problems and therefore have a greater need for therapy for bipolar-1-disorder than for bipolar-2-disorder, where depression tends to take centre stage. This could also explain why there are more studies on patients in a manic episode than in a depressive episode among the studies analysed (Fig. 5). However, the latter could also be due to the fact that lithium is used both for phase prophylaxis and for the treatment of acute bipolar mania. In contrast, other drugs like carbamazepine, quetiapine, or olanzapine are preferred for the treatment of acute bipolar depression, but lithium-monotherapy, however, is not recommended (DGBS e.V. and DGPPN e.V.: S3 guideline on the diagnosis and treatment of bipolar disorders. Long version, 2019; last call: 19.12.2023).

### Short study periods

Lithium takes approximately 4–10 days to take effect (https://www.msdmanuals.com/de-de/heim/psychische-gesundheitsstörungen/affektive-störungen/bipolare-störung#:~:text=Da%20Lithium%204%20to%2010,faster%20acting%20drug%20like%20z; last accessed: 16.11.23). The patients who were included in the analysed studies had mostly been on lithium therapy for a longer period of time, already. It can therefore be assumed that the onset of the lithium effect was already present at the start of the study. Nevertheless, it should be taken in account critically that the majority of the studies only lasted for a period of < 20 weeks (Fig. 6). This is because bipolar disorder is an illness that accompanies patients for many years of their lives. The episodes of depression, mania, or hypomania can last for several weeks to months at a time. In between, patients repeatedly experience phases of normal mood (https://dgbs.de/bipolare-stoerung/verlauf; last accessed: 16.11.23). Therefore, long-term studies are more suitable for reliably assessing the effect of various drugs during the course of the illness. Lithium is mainly used as a long-term phase prophylaxis of bipolar disorder. Due to the small number of studies that lasted over a period of > 40 weeks (*N* = 12 of *N* = 59), this represents a significant limitation in the assessability of lithium in long-term use.

### Economic and scientific interest in lithium

Approximately 0.5% of the global population suffer from bipolar disorder (https://de.statista.com/statistik/daten/studie/1078901/umfrage/anteil-der-weltbevoelkerung-mit-bipolarer-stoerung/#:~:text=In%20year%202019%20suffered%20worldwide,at%20about%200%2C5%20percent; last call: 06.12.2023). The fact that only eight different countries, including only two European countries, conducted studies on lithium therapy for bipolar disorder (Fig. 7) is surprising, as more international interest would be expected due to the global relevance of bipolar disorder.

**Fig. 9:**
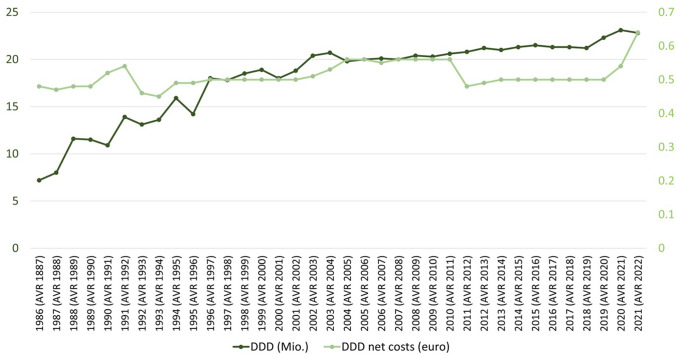
Depiction of the DDD costs and DDD in Germany over time. Numbers were taken from the Drug Prescription Report (Arzneiverordnungsreport, AVR) from 1986-2021

The few companies that supported the studies financially (Fig. 8) produce the additional medication for the respective study. It can be assumed that the companies did not support the studies out of financial interest in lithium. The low interest of the pharmaceutical industry in lithium could be explained by the low cost of lithium, as illustrated by the DDD costs in Germany (Fig. 9). The DDD costs were taken from the “Drug Prescription Reports” (Arzneiverordnungsreport, AVR) from Germany.

The rising trend in DDD in Germany over time clearly shows that interest in lithium therapy is increasing (Fig. 9). This may be due to the poorer efficacy of alternative drugs. Based on the DDD for 2021 and a 12-month prevalence of 0.29%, it can be calculated that only approx. 29% of patients with bipolar disorder covered by statutory health insurance have received lithium. This indicates a gap in the treatment of bipolar disorder. This is also supported by a study that investigated the percentage distribution of drugs used in patients with bipolar disorder. The use of antidepressants in particular is popular. In contrast, the use of lithium was low. In addition, during the decline in lithium use, an increase in prescriptions of other mood stabilisers, such as quetiapine, valproic acid, and lamotrigine, could be seen (Poranen et al. 2022).

### Lithium monotherapy

*N* = 3 of *N* = 59 studies analysed dealt with lithium-monotherapy. One of them dealt with the treatment of children (Fig. 3). In patients with bipolar-1-disorder, several clinical predictors were identified that are significantly associated with non-response to lithium. These include pre-existing anxiety symptoms, functional impairment, negative life events, migraines, suicidal thoughts/attempts, mixed episodes, chronic disease course, and personality disorders (Lin et al. 2021). Even though lithium is the gold standard for the treatment of bipolar disorder, not all patients respond to therapy. If clinical predictors of non-response to lithium were known, a decision could be made before starting treatment with lithium as to whether such treatment makes sense or whether alternative drugs should be chosen. It is therefore all the more important that further studies are planned in order to establish a reliable link between predictors and non-response (Lin et al. 2021).

An earlier study investigated predictors for the response to lithium, risperidone or valproic acid. However, this study only showed that children with ADHD responded better to risperidone. No predictors could be identified for lithium (Vitiello et al. 2012). A statistically significant improvement in lithium-induced tremor was achieved by switching from lithium IR to lithium PR (Pelacchi et al. 2022). This could be a starting point for improving other common lithium-induced adverse drug reactions.

### Lithium vs. placebo/drug

The results of this subgroup are summarised in Table 1.

**Table 1:**
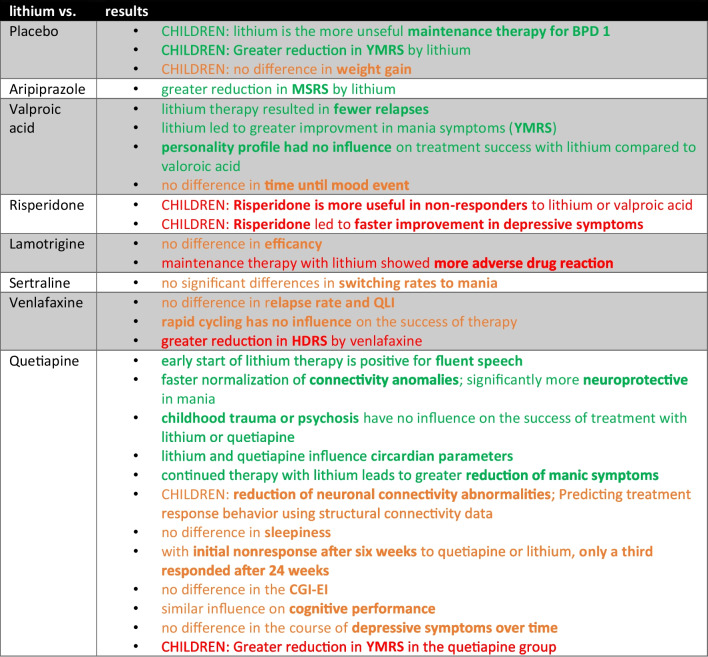
Depiction of the compared medications to lithium and some study results; marked green, lithium was superior; marked orange, no difference between lithium and compared medication; marked red, compared medication performed better than lithium

Overall, lithium is superior to quetiapine and valproic acid in particular. In comparison with lamotrigine, sertraline, and venlafaxine, lithium therapy appears to be equal (Table 1). Both included studies comparing lithium with placebo observed paediatric patients, likewise the studies on risperidone (*N* = 2).

### Lithium vs. aripiprazole for the treatment of manic symptoms

One of the 59 studies compared aripiprazole with lithium. Lithium was superior to aripiprazole both in improving mania symptoms and in terms of adverse drug reactions (Shafti 2018). However, the results of this study should be viewed critically, as only Iranian male patients were included in the study. This indicates that there is a lack of ethnic and gender variability in the study population. A previous comparative study of aripiprazole and lithium showed that aripiprazole led to a more rapid reduction in YMRS than lithium in patients with bipolar-1-disorder. After 12 weeks, however, the reduction in YMRS was similar for lithium and aripiprazole (Keck et al. 2009).

### Lithium vs. valproic acid for the treatment of manic symptoms

*N* = 7 of *N* = 59 studies compared lithium with valproic acid; *N* = 2 of these examined children. Both lithium and valproic acid are well tolerated and improve the symptoms of mania, with lithium causing a greater reduction in YMRS than valproic acid (Young et al. 2017). Patients on lithium therapy did not relapse for significantly longer compared to patients on valproic acid therapy (Peselow et al. 2016). The personality profile of patients with psychotic mania had an influence on the success of treatment with valproic acid, but not on the success of treatment with lithium (Akbarzadeh et al. 2022). There was no significant difference between the treatment groups in terms of sedation (Young et al. 2017). Similarly, no significant difference was found between the two therapies in terms of time to onset of a mood event (Kang et al. 2020). Overall, lithium and valproic acid showed similar efficacy, although lithium may be beneficial in patients with psychotic mania and personality disorder. Lithium may also be the better alternative to valproic acid in terms of improving mania symptoms and preventing relapses. Only one of the studies analysed reported a significantly higher discontinuation rate in the lithium group than in the valproic acid group (Gao et al. 2020).

### Lithium vs. lamotrigine

The most common adverse drug reactions from lithium therapy included cognitive slowing, impaired memory, increased thirst, boredom, tremors, and amnesic aphasia (Parker et al. 2021). An earlier study, which lacked a placebo control and blinding, showed similar efficacy of lithium and lamotrigine. Lithium therapy was associated with more adverse drug reactions than lamotrigine-therapy, but this did not lead to increased dropouts in the lithium group (Suppes et al. 2008).

### Lithium vs. sertraline

*N* = 1 of *N* = 59 studies analysed contrasted lithium-monotherapy with sertraline-monotherapy. There was no difference in the rates of mood change from depression to mania between sertraline and lithium (Altshuler et al. 2017).

### Lithium vs. venlafaxine for the treatment of depressive episodes

*N* = 4 of N = 59 analysed studies compared lithium-monotherapy with venlafaxine-monotherapy. In acute therapy, a greater reduction in depressive symptoms (HDRS17) was achieved with venlafaxine (Amsterdam et al. 2016). There was no difference between lithium and venlafaxine in terms of depressive relapse during continued therapy (Amsterdam et al. 2015). Patients also showed no significant difference between the two therapies in terms of quality of life (QLI) (Lorenzo-Luaces & Amsterdam 2018). Patients with rapid cycling also showed no significant difference in treatment response (> 50% reduction in HDRS17 compared to the start of the study) and in the prevention of depressive relapse compared to patients without rapid cycling (Lorenzo-Luaces et al. 2016).

### Lithium vs. quetiapine

Fifteen out of 59 studies analysed lithium-monotherapy with quetiapine-monotherapy. Three of the studies treated patients of paediatric age. Lithium was superior to quetiapine in some respects. However, there are also some points where no difference was found between lithium and quetiapine (Table 1).

### Lithium vs. quetiapine in patients with manic episode

Since patients with bipolar disorder often also suffer from cognitive impairment, it is important to know the influence of different drugs on the relevant brain structures. In adult patients, lithium improved connectivity in the brain better than quetiapine and it showed a positive influence on patients’ fluency of speech (Daglas et al. 2016; Dandash et al. 2018). Lithium revealed a larger neuroprotective effect than quetiapine but the effect on cognitive performance was similar (Berk et al. 2017b; Daglas et al. 2016). It is discussed whether the efficacy of lithium is due to its influence on various neuronal structures. An earlier study revealed that patients undergoing lithium therapy exhibited an increase in grey matter (Lyoo et al. 2010). Continued therapy with lithium led to a greater improvement in manic symptoms and, in contrast to quetiapine therapy, lithium therapy did not result in a deterioration of cardiometabolic parameters (Berk et al. 2017a; Kuperberg et al. 2022).

### Lithium vs. quetiapine in patients with depressive episode

Both lithium and quetiapine have an effect on circadian parameters, with quetiapine leading to a significant delay in the acrophase (Hwang et al. 2017). Pre-existing childhood trauma or psychotic symptoms had no influence on treatment outcomes with lithium or quetiapine (Caldieraro et al. 2018; Wrobel et al. 2022). Lithium and quetiapine did not differ with regard to the improvement of the CGI-EI and the time course of depressive symptoms (Behrendt-Møller et al. 2019; Nierenberg et al. 2016). Similarly, sleepiness occurred equally in both treatment groups (Gao et al. 2019). Among the patients who were non-responders to lithium or quetiapine after 6 weeks, only one-third demonstrated a response to the medication after 24 weeks. This highlights the need for the search for treatment alternatives for patients with non-response to lithium (Köhler-Forsberg et al. 2021).

### Lithium + adjunctive therapy

**Fig. 10:**
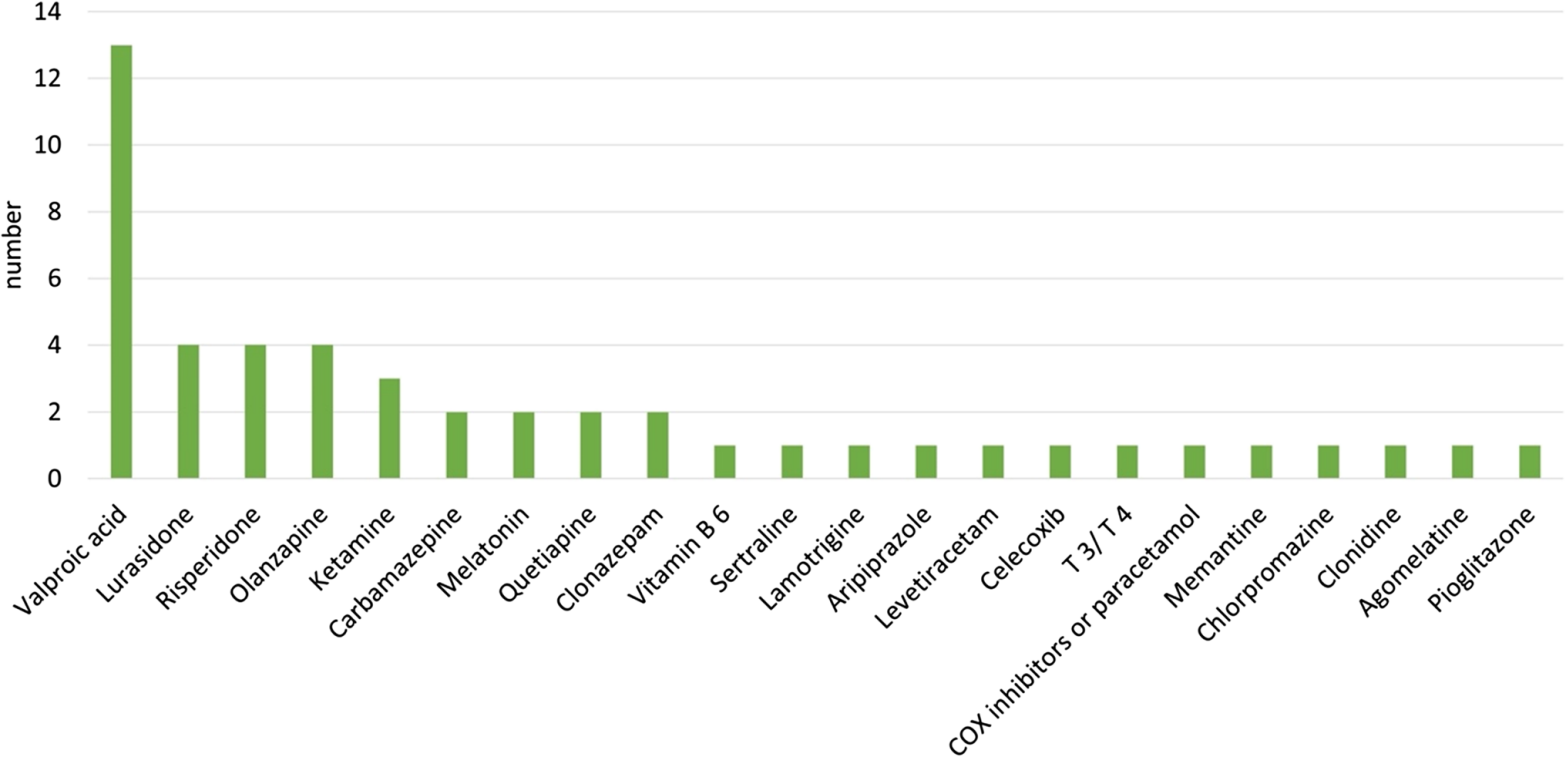
Type of adjunctive medication

As can be seen from the summary of this subgroup in Table 2, of the 22 additional therapies analysed (Fig. 10), a total of *N* = 14 showed a predominantly positive effect and only one study showed a predominantly negative effect. In some studies, however, there was no direct comparison with lithium-monotherapy. Thus, in these cases, it cannot be assessed whether the effect would also have occurred with monotherapy. Only one of the studies in the subgroup “lithium + adjunctive therapy” deals with the treatment of adverse drug reactions. Given the diverse adverse drug reactions associated with lithium therapy, it would have been expected that some of the adjunct therapy studies would have focused more on managing adverse drug reactions.

**Table 2:**
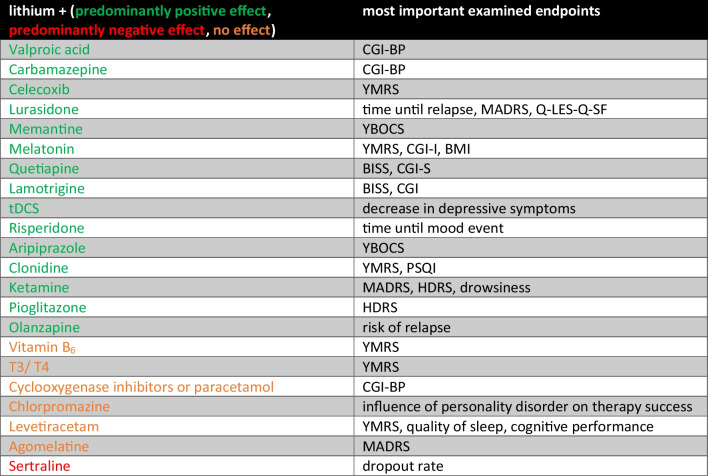
Depiction of adjunctive medication to lithium and the most important endpoints examined; marked green, adjunctive medication with predominantly positive effect; marked orange, adjunctive medication with no effect; marked red, adjunctive medication with predominantly negative effect

### Additional medications with a predominantly positive effect in maintenance therapy

Additional therapy with carbamazepine or valproic acid was well tolerated. Weight gain was observed with valproic acid, in contrast to a slight weight loss with carbamazepine (Missio et al. 2019). However, there was no comparison with the monotherapy, which is why no statement can be made as to whether the additional therapy is more effective or better tolerated than the monotherapy. A combination of lithium and lurasidone resulted in an extension of the time until the occurrence of a mood event compared to the respective monotherapy (Calabrese et al. 2017).

### Additional medications with a predominantly positive effect on depressive symptoms

For patients with depressive symptoms, the use of transcranial direct current stimulation could be an effective additional therapy to drug treatment (Mardani et al. 2021). A greater improvement in depressive symptoms (HDRS) than with placebo could also be achieved through additional therapy with pioglitazone (Zeinoddini et al. 2015). In addition, the quality of life (measured with Q-LES-Q-SF) of the patients was improved by the lurasidone add-on therapy (Rajagopalan et al. 2016). With regard to the improvement of depressive symptoms (MADRS scores), an improvement was observed with lurasidone add-on therapy, but this was not significant (Sajatovic et al. 2016; Suppes et al. 2016). Additional administration of ketamine reduced depressive symptoms and fatigue in patients who were resistant to treatment (Ionescu et al. 2015; Saligan et al. 2016). However, a direct comparison with lithium-monotherapy is also lacking here. Serum lithium or valproic acid concentrations had no influence on the antidepressant effect of ketamine (Xu et al. 2015). The addition of lamotrigine or quetiapine to lithium had positive effects compared to lithium-monotherapy in terms of reduction in BISS and CGI-S values as well as a significant increase in survival time (Gao et al. 2020; Valdes et al. 2019).

### Additional medications with a predominantly positive effect on manic symptoms

For a period of 24 weeks, additional treatment with risperidone or olanzapine, compared to lithium-monotherapy, reduced the probability of manic episodes and the risk of relapse. Beyond the 24-week period, the additional therapy had no further effect (Kang et al. 2020; Yatham et al. 2016a). This study clearly indicates that it is very important to observe the effectiveness of the various drugs over a longer period of time in bipolar disorder. A greater reduction in mania symptoms (YMRS) was achieved with both adjuvant celecoxib and clonidine in addition to lithium therapy than with lithium-monotherapy, with adjuvant clonidine also improving sleep quality of patients (Ahmadpanah et al. 2022; Mousavi et al. 2017). In manic patients with additional obsessive-compulsive symptoms, additional therapy with both memantine and aripiprazole yielded a greater reduction in YBOCS values than in patients without additional therapy (Sahraian et al. 2017; Sahraian et al. 2018). Add-on therapy with melatonin improved mania symptoms compared to lithium-monotherapy (Moghaddam et al. 2020).

Melatonin has a pronounced first-pass effect and its bioavailability is around 15%. In addition, it has a short renal elimination half-life of only 3.5–4 h (https://www.gelbe-liste.de/wirkstoffe/Melatonin_50325#Pharmakokinetik; last call: 07.12.2023). Due to these aspects, a positive effect of melatonin in the treatment of bipolar disorder must be viewed critically, as larger quantities of melatonin had to be consumed throughout the day to achieve a lasting effect. At a dose of 6 mg/day, it cannot be assumed that a sufficient serum concentration will be achieved to demonstrate efficacy.

### Additional medication with a predominantly negative effect

Add-on therapy with sertraline increased discontinuations compared to lithium-monotherapy. There was no difference between monotherapy and add-on therapy with regard to the risk of a change in mood (Altshuler et al. 2017).

### Additional medication without effect

Six of 22 additional medications showed no significant on lithium therapy (Table 2). Comorbid psychosis had no influence on the treatment outcome when treated with lithium and adjuvant chlorpromazine or olanzapine (Hasty et al. 2022). Also the additional intake of cyclooxygenase inhibitors or paracetamol had no influence on the treatment outcome (Köhler-Forsberg et al. 2017). This implies that analgesic, antiphlogistic, and antipyretic therapy is possible despite lithium therapy. However, due to a possible increase in lithium plasma concentrations when taking cyclooxygenase inhibitors, these should be closely monitored when taken currently. Neither adjuvant levetiracetam nor adjuvant vitamin B_6_ to lithium significantly improved mania symptoms or sleep quality (Badrfam et al. 2021; Keshavarzi et al. 2022). Additional medication with agomelatine did not achieve a significant improvement in MADRS scores in depressed patients (Yatham et al. 2016b). With adjuvant T3 or T4, patients spent more time in a normal mood than with placebo (Walshaw et al. 2018).

### Paediatric patients

**Fig. 11:**
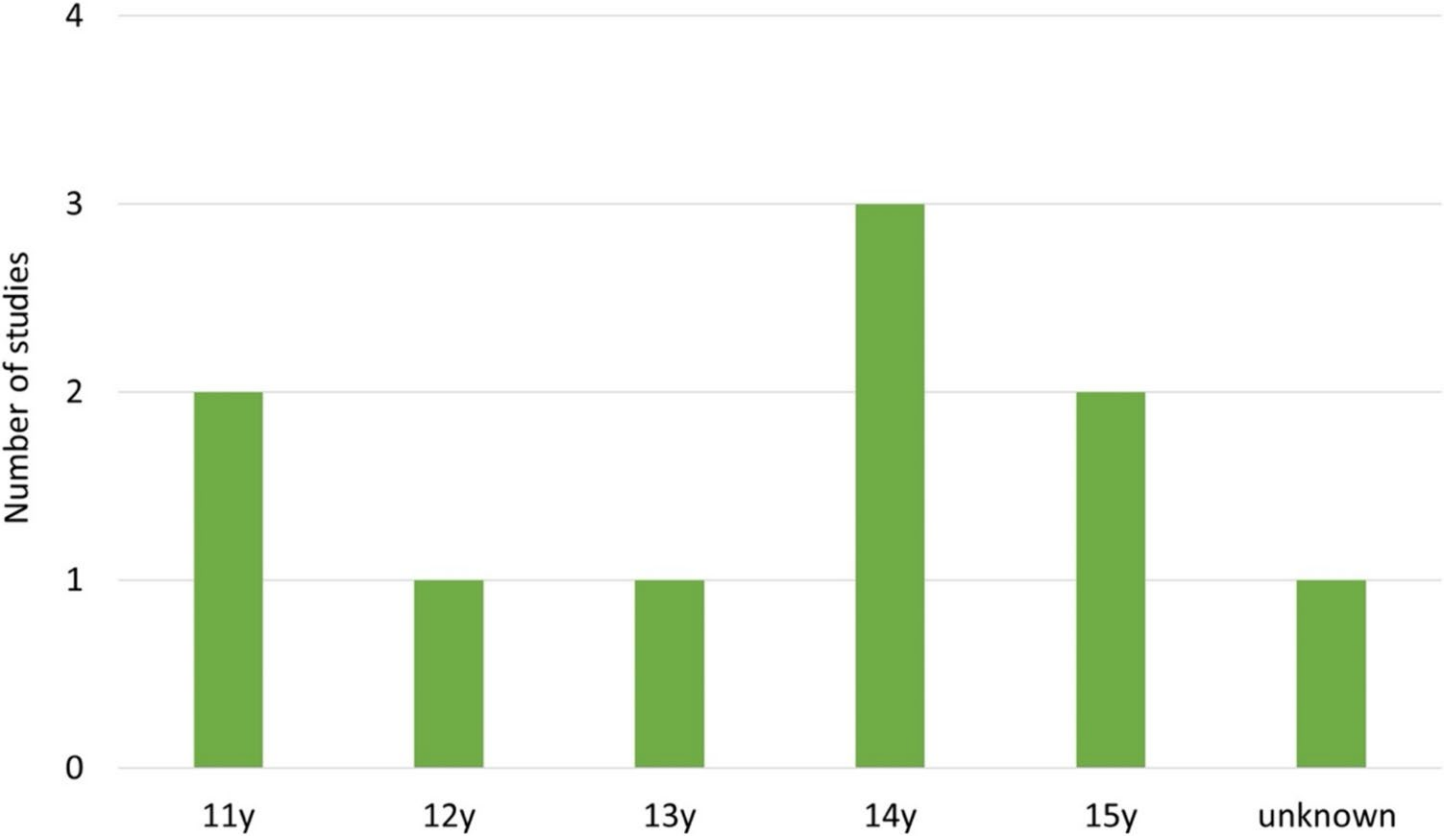
Mean age of paediatric study participants

The average age of the children in the studies was 11–15 years (Fig. 11). Forty percent of the diagnoses are made between the ages of 17 and 21. For this reason, the age chosen in the studies may be somewhat too young. In addition, the diagnosis of bipolar disorder in children is controversial, as there are several differential diagnoses that are associated with similar symptoms and behaviour in children such as ADHD, depression, early-phase schizophrenia, social behaviour disorders, and anxiety disorders (https://www.springermedizin.de/emedpedia/psychiatrie-und-psychotherapie-des-kindes-und-jugendalters/bipolare-stoerungen-im-kindes-und-jugendalter?epediaDoi=10.1007%2F978-3-662-49289-5_102; last call: 19.12.2023).

**Fig. 12:**
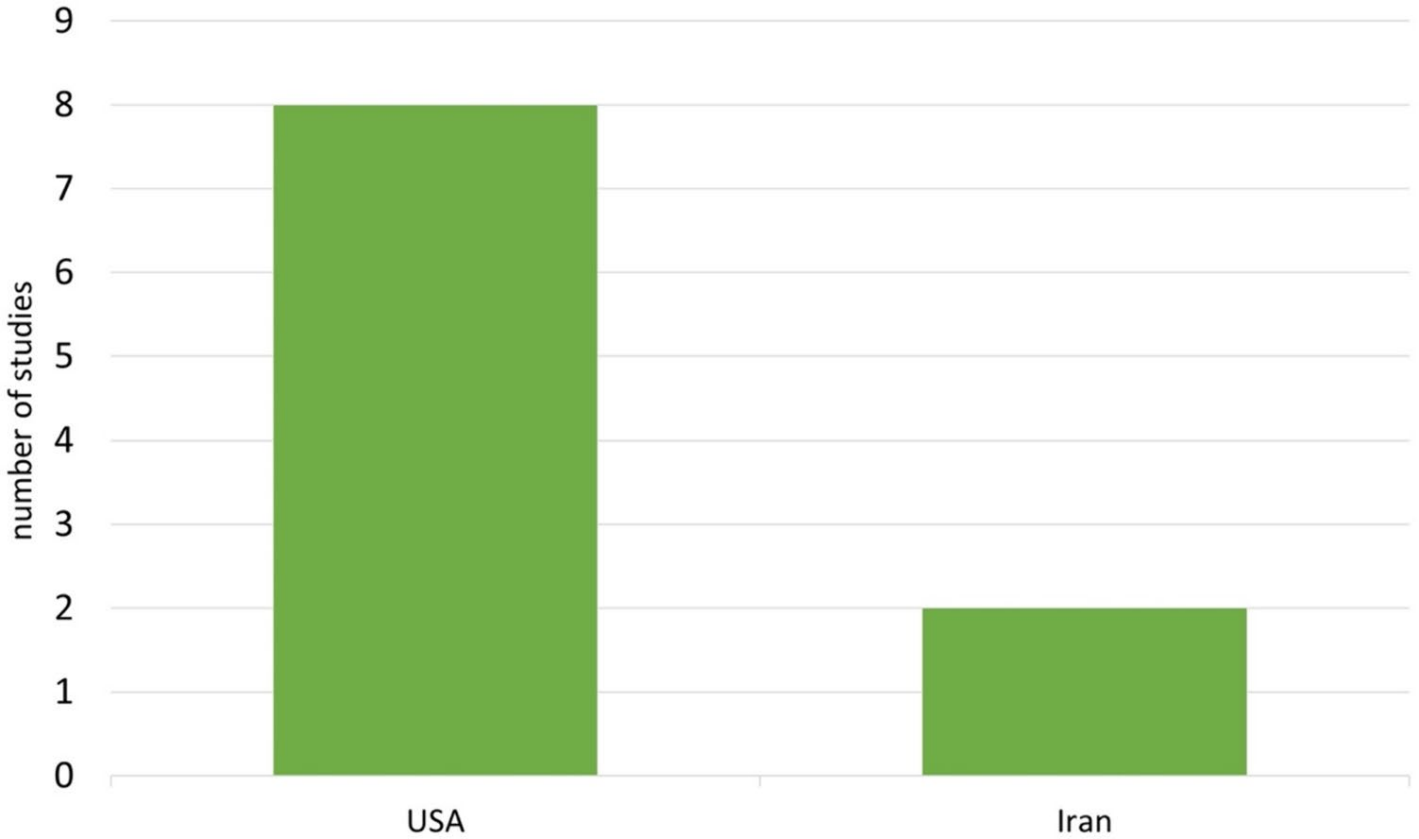
Country of study conduct

There are more diagnoses of childhood bipolar disorder in the USA than in European countries (https://www.springermedizin.de/emedpedia/psychiatrie-und-psychotherapie-des-kindes-und-jugendalters/bipolare-stoerungen-im-kindes-und-jugendalter?epediaDoi=10.1007%2F978-3-662-49289-5_102; last accessed: 19/12/2023). Thus, this disorder is interpreted differently in various countries. The increased diagnosis of childhood bipolar disorder in the USA is also reflected in the studies analysed. Eight of 10 studies were conducted in the USA, none in a European country (Fig. 12). The question therefore arises as to what extent bipolar disorder is a relevant illness in children and whether the prevalence is overestimated due to misdiagnosis or underestimated due to a lack of diagnosis.

### Studies on paediatric patients with manic or mixed episode

There was no significant difference between lithium and placebo in terms of weight gain (Findling et al. 2015). Lithium was superior to placebo in reducing manic symptoms (Findling et al. 2019). This aspect in particular was normalised lithium in comparison to other drugs that often present weight gain as adverse drug reaction. Melatonin as an add-on to lithium showed an almost significant reduction in weight gain compared to placebo (Mostafavi et al. 2017). However, this study was not only conducted on manic patients, but on patients in every phase of the disease. Two previously mentioned studies showed that lithium did not lead to any significant weight gain compared to placebo (Findling et al. 2015, 2019). Due to its pharmacokinetics, melatonin would have to be dosed very highly to show a weight-reducing effect. At this point, studies with weight loss drugs such as lamotrigine might be useful.

The pharmacokinetics of lithium in adults are well known. The pharmacokinetics in children are similar to those in adults, taking body size into account (Landersdorfer et al. 2017). Thus, therapeutic drug monitoring is also indicated in children. Quetiapine achieved a greater reduction in manic symptoms and a higher response rate in children compared to lithium (Patino et al. 2021). Quetiapine could therefore lead to better therapeutic success than lithium in paediatric patients with bipolar-1-disorder in a manic or mixed episode. But combination of lithium and celecoxib resulted in a significantly greater reduction in YMRS compared to placebo in patients with acute mania (Mousavi et al. 2017). Risperidone showed the highest response rates as continuation therapy in paediatric non-responders to lithium, valproic acid, or risperidone (Walkup et al. 2015). These results demonstrate that, in addition to lithium, risperidone and quetiapine should be considered as treatment options for paediatric patients.

Altered brain connectivity was observed in children with bipolar disorder. Both lithium and quetiapine normalised connectivity. It was also possible to predict whether the patients would respond to treatment based on their cognitive structures (Lei et al. 2021; Li et al. 2022). In the future, this could be an approach to determine which medication is most suitable for the respective patient before starting therapy.

### Studies on paediatric patients with depressive episode

Risperidone additionally achieved better results than lithium in children with regard to depressive symptoms (Salpekar et al. 2015).

### Methodological criticism

**Fig. 13:**
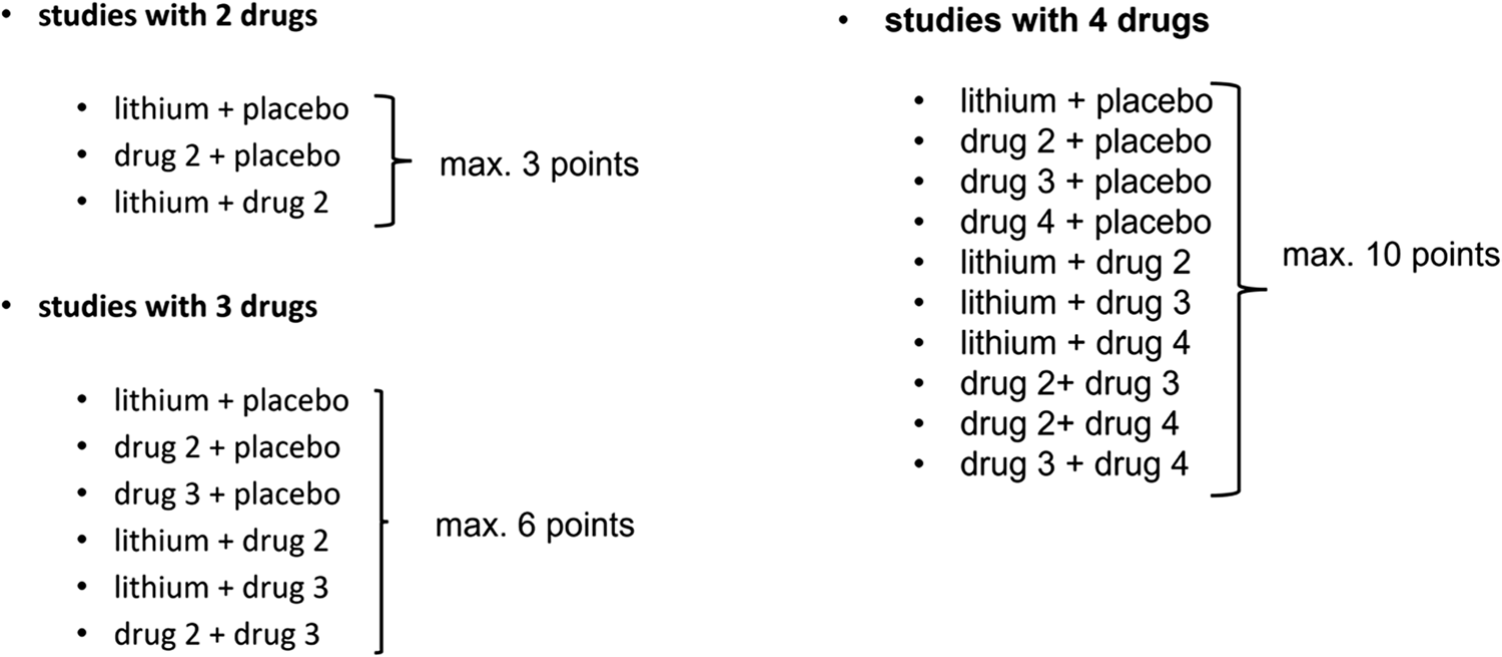
Explanation of the point system for methodology evaluation of the studies with adjunctive therapy

The methodology of the studies from the subgroup “lithium + adjunctive therapy” must be viewed critically. The comparison of several drugs can easily lead to additive, overadditive, synergistic, or even subtractive effects not becoming visible. When comparing lithium + valproic acid with lithium + carbamazepine, a total of six treatment groups would have to be compared against each other (lithium + placebo, valproic acid + placebo, carbamazepine + placebo, lithium + valproic acid, lithium + carbamazepine, carbamazepine + valproic acid) in order to avoid effect errors. The methodology of the individual studies was assessed using a point system. Studies with two different drugs were awarded a maximum of 3 points, studies with three different drugs a maximum of 6 points (see example study), and studies with four different drugs a maximum of 10 points (Fig. 13).

With the exception of one study, none of the 27 studies received full points. In the group of studies with two different drugs, ten studies scored 2/3 points and one study scored 3/3 points (Table 3). In the group of studies with three different drugs, five studies received 2/6 points, one study 3/6 points, and five studies 4/6 points (Table 3). In the studies with four different drugs, one study scored 4/10 points, two studies scored 6/10 points, and one study scored 7/10 points (Table 3). Not only the number and type of control groups are responsible for the quality of the study. In addition, duration of the study, the design of the study, and the number of participants are decisive. For this reason, Tables 3, 4, and 5 list these criteria in addition to the respective points achieved with regard to the control groups of the studies. On a positive note, 26 studies were conducted in the design of a randomised clinical trial and only 1 as a clinical trial.

**Table 3:** Depiction of the evaluation of the methodology of the studies with 2 different drugs

Adjunctive therapy	Study design	Number of patients	Duration of the study	Points based on study control groups	Reference
Vitamin B_6_	RCT	50	8 w	2/3	Badrfam et al. 2021
Melatonin	RCT	60	6 w	2/3	Moghaddam et al. 2020
tDCS	RCT	30	12 w	2/3	Mardani et al. 2021
Aripiprazole	RCT	56	8 w	2/3	Sahraian et al. 2018
Levetiracetam	RCT	72	4 w	2/3	Keshavarzi et al. 2022
Celecoxib	RCT	42	8 w	2/3	Mousavi et al. 2017
Melatonin	RCT	48	12 w	2/3	Mostafavi et al. 2017
Memantine	RCT	38	16 w	2/3	Sahraian et al. 2017
Clonidine	RCT	70	4 w	2/3	Ahmadpanah et al. 2022
Pioglitazone	RCT	48	6 w	2/3	Zeinoddini et al. 2015
Sertraline	RCT	142	16 w	3/3	Altshuler et al. 2017

**Table 4:** Depiction of the evaluation of the methodology of the studies with 3 different drugs

Adjunctive therapy	Study design	Number of patients	Duration of the study	Points based on study control groups	Reference
Valproic acid or carbamazepine	RCT	64	2 y	2/6	Missio et al. 2019
Risperidone	RCT	93	1 y	2/6	Valdes et al. 2019
Olanzapine or chlorpromazine	RCT	71	1.5 y	2/6	Hasty et al. 2022
Ketamine	CT	36	2 w	2/6	Xu et al. 2015
Ketamine	RCT	36	2 w	2/6	Ionescu et al. 2015
T3 or T4	RCT	32	16 w	3/6	Walshaw et al. 2018
Lurasidone	RCT	496	48 w	4/6	Calabrese et al. 2017
Cyclooxygenase inhibitors or paracetamol	RCT	482	24 w	4/6	Köhler-Forsberg et al. 2017
Lurasidone	RCT	356	6 w	4/6	Suppes et al. 2016
Agomelatine	RCT	344	1 y	4/6	Yatham et al. 2016b
Ketamine	RCT	36	2 w	4/6	Saligan et al. 2016

**Table 5:** Depiction of the evaluation of the methodology of the studies with 4 different drugs

Adjunctive therapy	Study design	Number of patients	Duration of the study	Points based on study control groups	Reference
Risperidone or olanzapine	RCT	159	1 y	4/10	Yatham et al. 2016a
Lurasidone	RCT	142	6 w	6/10	Sajatovic et al. 2016
Lurasidone	RCT	825	6 w	6/10	Rajagopalan et al. 2016
Risperidone or olanzapine	RCT	159	1 y	6/10	Kang et al. 2020
Quetiapine or lamotrigine	RCT	112	28 w	7/10	Gao et al. 2020

Overall, the analysis of the studies showed that a large number of add-on medications to lithium have a positive effect on the therapeutic outcome (Table 2). However, with the exception of one study, the various drugs and drug combinations were not adequately presented.

### Presentation of the studies with the largest number of participants

In the following, the studies (*N* = 10) with the largest number of participants (> 300 participants) are presented separately (Table 6). Due to the large number of participants, it can be assumed that the study results are of particularly high clinical relevance.

**Table 6:** Survey of the studies with > 300 participants

Initial diagnosis	Disease episode	Participants	Duration	Country of study conduct	Study design	Reference
BPD 1	Every phase	345	2 y	International	CT	Lin et al. 2021
BPD 1 or BPD 2	Every phase	482	48 w	USA	RCT	Wrobel et al. 2022
BPD 1 or BPD 2	Depression	592	24 w	USA	RCT	Köhler-Forsberg et al. 2021
BPD 1 or BPD 2	Depression	303	24 w	USA	RCT	Caldieraro et al. 2018
BPD 1 or BPD 2	Every phase	770	24 w	USA	RCT	Kuperberg et al. 2022
BPD 1 or BPD 2	Every phase	482	24 w	USA	RCT	Nierenberg et al. 2016
BPD 1	Every phase	496	48 w	USA	RCT	Calabrese et al. 2017
BPD 1	Depression	356	6 w	USA	RCT	Suppes et al. 2016
BPD 1	Depression	825	6 w	USA	RCT	Rajagopalan et al. 2016
BPD 1 or BPD 2	Every phase	482	24 w	USA	RCT	Köhler-Forsberg et al. 2017

Patients (*N* = 345) with bipolar-1-disorder were followed for up to 2 years. They received lithium-monotherapy during this time. Eighty-eight of 345 patients showed a good response to therapy and had no relapse or showed remission, while 101 patients experienced treatment failure. Clinical predictors of non-response to lithium could be found. These include pre-existing anxiety symptoms, cognitive dysfunction, negative life events, migraines, suicidal thoughts/attempts, mixed episodes, chronic disease progression, and personality disorders. However, further studies are needed to establish a reliable link between predictors and non-response (Lin et al. 2021). In contrast, neither childhood trauma nor psychotic symptoms had an influence on the success of therapy with lithium or quetiapine (Caldieraro et al. 2018; Wrobel et al. 2022). No difference in therapy success could be found between patients (*N* = 482) with or without childhood trauma after 24 weeks of treatment (Wrobel et al. 2022).

Elevated cholesterol concentrations at the start of therapy also do not have any influence on the treatment response of lithium or quetiapine. However, elevated cholesterol and LDL concentrations are associated with more severe depression. In contrast to quetiapine-therapy, lithium therapy did not result in a deterioration of cardiometabolic parameters (Kuperberg et al. 2022).

Lithium and quetiapine did not differ in terms of improvement in CGI-EI and the time course of depressive symptoms. A total of 482 patients with bipolar-1- or bipolar-2-disorder were observed over a period of 6 months (Nierenberg et al. 2016).

In another study, of a total of 592 participants, 34% (*N* = 199) responded to therapy with lithium or quetiapine after 2 weeks. However, of the remaining 393 participants, 46% responded after 24 weeks. In total, a response was observed in 380/592 participants. In addition, patients who received additional antidepressants had the lowest response rates to lithium or quetiapine among all participants (Köhler-Forsberg et al. 2021).

Adjunctive therapy with lurasidone was investigated in 1,677 patients in three studies. A combination of lithium with lurasidone led to an increase in the time until a mood event occurred compared to the respective monotherapy. Study participants were randomised to receive either lithium-monotherapy, lurasidone-monotherapy, or lithium with lurasidone adjunctive therapy for 28 weeks. There were only minor effects on the weight of the patients and their metabolic parameters (Calabrese et al. 2017). A positive effect of this combination therapy was also the improvement of the quality of life of the patients (measured by Q-LES-Q-SF). However, in this study with *N* = 825 patients, lurasidone-monotherapy also led to this result (Rajagopalan et al. 2016). In terms of depressive symptoms (MADRS scores), improvement was observed with lurasidone adjunctive therapy, which was not significant after week 6. Adverse drug reactions of lurasidone were akathisia, somnolence, and extrapyramidal symptoms (Suppes et al. 2016). In all three studies, a combination therapy of lithium and lurasidone showed positive results compared to lithium-monotherapy for different endpoints, so that due to the large number of participants, a strong significance can be assumed here. Therefore, further studies with longer investigation periods seem to be very useful here.

The additional intake of cyclooxygenase inhibitors or paracetamol in addition to lithium was investigated in *N* = 482 patients. It had no influence on the treatment outcome. It was not important whether cyclooxygenase inhibitors and paracetamol were taken in combination or individually. Over a period of 6 months, the influence on CGI and BISS was assessed (Köhler-Forsberg et al. 2017).

Of the 10 studies mentioned, 9 were conducted as randomised clinical trials and 1 as a clinical study (Table 6). However, only 2 studies lasted > 40 weeks (Table 6). Thus, even on the basis of the higher-quality studies, no clear statement can be made about the long-term use of lithium. With regard to the inclusion criteria of the participants, both patients with bipolar-1- and patients with bipolar-2-disorder were included in six studies. Four studies included only patients with bipolar-1-disorder (Table 6). Looking at the phase of illness of the study participants, 6 studies included patients regardless of phase, and studies only included patients who were in a depressive episode (Table 6). Patients with bipolar-2-disorder and/or mania were not evaluated separately in a large study. Nine of the 10 studies were conducted in the USA (Table 6).

### Comparison of the present study with two recent reviews

One review focusses on the efficacy of lithium in acute mania, acute bipolar depression, in maintenance treatment, and in the treatment of other issues. A total of 307 studies from the MEDLINE database up to August 2002 were included. The monotherapy studies and combination therapy studies were also assessed separately. In conclusion, this study showed that lithium was effective against a wide range of clinical problems of bipolar disorder. The safety and tolerability of lithium was not assessed in this study (Fountoulakis et al. 2022).

The second study summarises 39 randomised controlled trials from the MEDLINE, CENTRAL, EMBASE, PsycINFO, and ClinicalTrials.gov databases up to April 2022. Randomised controlled trials comparing oral antimanic monotherapy with placebo in patients with acute mania were included. The variability of the improvement in manic symptoms, the improvement in manic symptoms, and the acceptance of the therapy were analysed. It was shown that risperidone, olanzapine, aripiprazole, cariprazine, ziprasidone, lithium, quetiapine, and haloperidol were associated with less variability and better efficacy than placebo. In addition to these eight drugs, asenapine also resulted in better efficacy than placebo (Hsu et al. 2022).

## Conclusions

The pharmaceutical industry has little interest in lithium. This may be due to the low cost of lithium. In addition, just around 29% of patients with bipolar disorder covered by statutory health insurance in Germany were prescribed lithium in 2021, which indicates a gap in care. Some clinical aspects are predictors for the response to lithium therapy and thus facilitate the treatment decision. An improvement in lithium-induced tremor was achieved by switching from lithium-IR to lithium-PR. Lithium was superior to aripiprazole, valproic acid, and quetiapine in terms of improving manic symptoms. Lithium therapy led to a lower relapse rate compared to valproic acid therapy. Lithium was more neuroprotective than quetiapine.

Fourteen of the 22 add-on therapies to lithium showed a predominantly positive effect on the treatment outcome compared to lithium-monotherapy. Exclusively, the add-on therapy with sertraline led to a higher rate of study discontinuations than lithium-monotherapy. Some of the additional drugs analysed showed no effect on the treatment outcome. These included vitamin B_6_, chlorpromazine, levetiracetam, and agomelatine. Additional therapy with cyclooxygenase inhibitors or paracetamol also revealed no effect on the treatment outcome. This leads to the conclusion that patients with lithium therapy can receive analgesic, antiphlogistic, and antipyretic therapy under close monitoring of the lithium plasma concentration. However, the methodology in most studies on adjunctive therapy is flawed, which is why the positive effect in comparison to monotherapy must be viewed critically.

Only one of the studies analysed was discontinued due to adverse drug reactions during lithium therapy. In children, lithium did not cause any relevant weight gain compared to placebo. In addition, it may be possible to draw a prediction about the response to therapy based on cognitive structures. Risperidone led to a faster improvement in depressive symptoms in children and showed better therapeutic success in non-responders to lithium, risperidone, or valproic acid. Quetiapine-therapy resulted in a greater improvement in mania symptoms and a higher response rate than lithium therapy.

## Future studies and limitations of the study

Overall, more long-term studies on the treatment of bipolar disorder are required to make a conclusion concerning the efficacy and safety of the various drugs during the course of the illness. Due to the small number of studies with a duration of > 40 weeks, this analysis is only possible to a limited extent. Clinical predictors for a response to lithium could be a great help in making treatment decisions. For this reason, further studies are needed. The improvement of lithium-induced tremor by switching to lithium-PR may represent an approach for improving adverse drug reactions. Further studies are also essential on treatment alternatives for non-responders to lithium. Since this analysis could not find a reason why there were more studies on patients in a manic episode than on patients in a depressive episode in the period 2015–2022, this also shows the need for further studies to better assess lithium therapy in depressive episodes. 

## Data Availability

All source data for this study are available upon reasonable request.

## Abbreviations

ADHD: Attention-deficit hyperactivity disorder
BISS: Bipolar Inventory of Symptoms Scale
BMI: Body mass index
BPD 1: Bipolar disorder 1
BPD 2: Bipolar disorder 2
CGI: Clinical Global Impression
CGI-BP: Clinical Global Impression - Bipolar Disorder
CGI-BP-I-D: Clinical Global Impression Scale for Depression
CGI-EI: Clinical Global Impression - Efficacy Index
CGI-I: Clinical Global Impression - Improvement Scale
CGI-S: Clinical Global Impression - Severity Scale
CDRS-R: Children’s Depression Rating Scale - Revised
CT: Clinical trial
DDD: Defined daily dose
fMRI: Functional magnetic resonance tomography
HDRS17: Hamilton Depression Rating Scale - 17 items
HDRS: Hamilton Depression Rating Scale
Lithium-IR: Lithium - immediate release
Lithium-PR: Lithium - prolongated release
MSRS: Manic State Rating Scale
MMSE: Mini Mental State Examination
MADRS: Montgomery-Asberg Depression Rating Scale
PSQI: Pittsburgh Sleep Quality Index
p-mGPCR antagonists: Antagonists at multiple G-protein-coupled receptors with pleiotropic effects
QLI: Quality of Life Index
Q-LES-Q-SF: Quality of Life Enjoyment and Satisfaction Questionnaire
RCT: Randomized clinical trial
T3/T4: Triiodothyronine/thyroxine
tDCS: Transcranial direct current stimulation
w: Week
y: Years
YMRS: Young Mania Rating Scale
YBOCS: Yale-Brown Compulsive Obsessive Scale
